# Blending of Low-Density Polyethylene and Poly(Butylene Succinate) (LDPE/PBS) with Polyethylene–Graft–Maleic Anhydride (PE–g–MA) as a Compatibilizer on the Phase Morphology, Mechanical and Thermal Properties

**DOI:** 10.3390/polym15020261

**Published:** 2023-01-04

**Authors:** Aina Aqila Arman Alim, Azizah Baharum, Siti Salwa Mohammad Shirajuddin, Farah Hannan Anuar

**Affiliations:** 1 Department of Chemical Sciences, Faculty of Science and Technology, Universiti Kebangsaan Malaysia, UKM, Bangi 43600, Selangor, Malaysia; 2 Polymer Research Center (PORCE), Faculty of Science and Technology, Universiti Kebangsaan Malaysia, UKM, Bangi 43600, Selangor, Malaysia; 3 Radiation Processing Technology, Malaysian Nuclear Agency, Kajang 43000, Selangor, Malaysia

**Keywords:** low-density polyethylene, poly (butylene succinate), compatibilizer, polymer blends, morphology, mechanical properties, thermal properties

## Abstract

It is of significant concern that the buildup of non-biodegradable plastic waste in the environment may result in long-term issues with the environment, the economy and waste management. In this study, low-density polyethylene (LDPE) was compounded with different contents of poly(butylene succinate) (PBS) at 10–50 wt.%, to evaluate the potential of replacing commercial plastics with a biodegradable renewable polymer, PBS for packaging applications. The morphological, mechanical and thermal properties of the LDPE/PBS blends were examined in relation to the effect of polyethylene–graft–maleic anhydride (PE–g–MA) as a compatibilizer. LDPE/PBS/PE–g–MA blends were fabricated via the melt blending method using an internal mixer and then were compression molded into test samples. The presence of LDPE, PBS and PE–g–MA individually in the matrix for each blend presented physical interaction between the constituents, as shown by Fourier-transform infrared spectroscopy (FTIR). The morphology of LDPE/PBS/PE–g–MA blends showed improved compatibility and homogeneity between the LDPE matrix and PBS phase. Compatibilized LDPE/PBS blends showed an improvement in the tensile strength, with 5 phr of compatibilizer providing the optimal content. The thermal stability of LDPE/PBS blends decreased with higher PBS content and the thermal stability of compatibilized blends was higher in contrast to the uncompatibilized blends. Therefore, our research demonstrated that the partial substitution of LDPE with a biodegradable PBS and the incorporation of the PE–g–MA compatibilizer could develop an innovative blend with improved structural, mechanical and thermal properties.

## 1. Introduction

Population growth, economic progress and demand for commodities have been the main driving forces for the production of plastic products in the last few decades, as they are lightweight, easily processed, durable and affordable [1]. Plastics are now a ubiquitous and indispensable part of modern society, which are used in various fields, including commerce, household, packaging and industries [2,3]. These single-use plastics, often known as disposable plastics, are used only once before being discarded or recycled [4]. According to global economic growth, by 2050, the amount of plastic waste generated will surpass 25 billion metric tonnes [5]. However, the widespread use of non-biodegradable plastic has become a considerable concern due to its adverse effects on the environment, human health and dependency on depleting fossil fuel resources [6]. The majority of conventional petroleum-based polymers are polyolefins, including polypropylene (PP), polystyrene (PS), polyvinyl chloride (PVC), polyethylene (PE) and polyethylene terephthalate (PET), because of their lightweight, superior mechanical properties and good resistance [7]. These polymers end up in landfill burial sites or are directly dumped into seas, where they persist for a long period of time as they are resistant to microbial attack, chemical and biological inertness, as a result of high molecular weight (M_w_) and hydrophobicity without the existence of functional groups [8,9]. PE can be divided into three categories: high-density polyethylene (HDPE), low-density polyethylene (LDPE) and linear low-density polyethylene (LLDPE) based on its density and branch content [10]. LDPE, a polymer with long- and short-chain branching, is well recognised for its strong mechanical properties, good water vapour barrier and thermal stability. Nevertheless, the continuous use of LDPE could lead to persistent issues with waste management and the environment [11,12]. The recycling of non-biodegradable plastics is a solution to this issue; moreover, this waste management is quite expensive and recycled products have poorer qualities than new products. In addition, it is difficult to totally recycle used food packaging as pollutants and/or chemicals can enter the food directly from the package [13]. Meanwhile, the total substitution of synthetic plastic with biodegradable polymers is an inefficient process since biodegradable polymers are costly and have low performance and a limited shelf life [14]. Therefore, manufacturing PE combined with biodegradable counterparts, including poly(butylene succinate) (PBS) [15], polyhydroxybutyrate (PHB) [16] and poly(lactic acid) (PLA) [17], has attracted considerable interest as a result of the potential to reduce the cost for large-scale applications and improving the properties of end-products and reducing the environmental issues.

Biodegradable polymers, also known as biopolymers, are prone to degrading naturally due to the action of microorganisms, which can transform them into water, carbon dioxide, biomass and methane [18]. According to their synthesis process, biodegradable polymers can be categorised into four classes: biomass products, from microorganisms, from biotechnology, and from petrochemical products [19]. Among the biodegradable aliphatic polyesters, PBS has good melt processability, excellent mechanical properties and thermal stability, comparable to those of the widely used PE and PP [20]. PBS is a compostable and biodegradable aliphatic polyester, which can be produced through the condensation of succinic acid and 1,4-butanediol [21]. PBS has been used in numerous fields, including biomedical, food packaging, agricultural and automotive [22]. The well-known drawbacks of PBS, such as high stiffness, low melt viscosity, poor impact resistance and increased production costs, have turned out to be the main disadvantages for wide applications in several industries [23]. Therefore, blending of polymers is one of the most economical ways to solve these problems related to neat PBS, as it will enhance the properties without sacrificing biodegradability. The use of a biodegradable polymer is helpful in terms of degradation, meaning that the biodegradable component will be eliminated by microorganisms, increasing the contact surface area between PE and the environment to which it is exposed. This will encourage more oxygen uptake and speed up the oxidation of PE chains. As polyethylene degrades, it undergoes the Norrish reaction that creates free radical, terminal vinyl groups and ketone groups and causes the main-chain scission of the polymer [24]. Then, free radicals and oxygen combine to form peroxy radicals, which ultimately transformed to peroxide. The compounds, e.g., alcohols, carboxylic acids, ketones, aldehydes or esters, can be produced during the decomposition process of peroxides [25]. Thus, the residual polymer matrix will be weakened and easily decomposed [26]. These partial replacements are more sustainable, with reduced carbon footprints and improved performance [27].

Polymer blending efficiently and economically provides desirable end products by improving their properties through combining two or more different polymers by physical mixing, with or without chemical interaction between them [28]. The primary drawback of combining LDPE with PBS is the poor of miscibility, since PBS is a polar polymer, which has oxygen-based groups and ester groups in its structure, whereas LDPE is a non-polar polymer. As a result, the poor interfacial adhesion among them causes inferior mechanical properties [29]. In fact, incompatibility in polymer blends leads to unstable morphology and weak mechanical properties, whereas compatibility in polymer blends demonstrates good properties [30]. The different M_w_ of polymer should be taken into consideration as it relates to the miscibility of the blends [31]. The existence of a surface field may cause segregation as a result of the surface’s differential coupling to one of the two components [32]. The phase segregation or even phase separation could occur in the system as the molecules with proportionally more chain ends (i.e., shorter chains) will be favoured at the interface to reduce the entropy penalty due to the different M_w_ [33]. The branched polymer is typically preferred to a surface in a blend of branched and linear polymers [34]. During melt blending, the compatibilizer agents move to the interface between the blend phases, which increases compatibility and enhances interfacial adhesion [35]. The incorporation of a compatibilizer, such as polyethylene–graft–maleic anhydride (PE–g–MA), has been proposed to ameliorate the lack of miscibility between the PBS and the LDPE matrix. Utilizing a compatibilizer is crucial in decreasing the domain sizes of the dispersed phase and enhancing the interfacial adhesion and, subsequently, mechanical properties.

Moreover, numerous studies have been published in recent years to improve the compatibility of the blend. For instance, Hemsri et al. [36] discussed the influence of PE–g–MA on the structural, mechanical and thermal properties of LDPE/poly(butylene adipate-co-terephthalate) (PBAT) films. The incorporation of a compatibilizer demonstrated more homogeneity and better compatibility with superior mechanical properties, resulting from greater interfacial adhesion between the LDPE and PBAT phase without affecting the thermal properties of the blends. Oner et al. [37] researched the inclusion of PE–g–MA, maleic–anhydride-modified ethylene propylene rubber (EPMgMAH) and ethylene maleic anhydride copolymer (PEMAH) for recycled polyethylene/thermoplastic starch (r-LDPE/TPS) blend modification. It can be concluded that PE–g–MA showed an excellent outcome on the tensile results among the others and is suitable for the packaging industry. Ferreira et al. [38] studied PLA/BioPE compatibilized with PE–g–MA. Compared to PLA, the binary blend has less properties due to immiscibility; thus, the addition of PE–g–MA promoted the interactions. As a result, properties increase in terms of mechanical and thermal stability. Nunes et al. [39] examined the impact of PE–g–MA on the morphological and thermal properties of PBAT/PLA blends. They demonstrated the function of PE–g–MA as a compatibilizer in enhancing the miscibility of PBAT/PLA blends. As described by Madhu et al. [40], they discovered that PE–g–MA served as an effective compatibilizer for the HDPE/poly(L-lactic acid) (PLLA) blends. The results proved that the compatibilizer promotes the better dispersion of PLLA particles in the matrix, thereby improving the mechanical resistance of the blends.

In the current study, polyethylene–graft–maleic anhydride (PE–g–MA) was employed as a compatibilizer and added into the LDPE/PBS blends to improve the interfacial adhesion between the LDPE matrix and PBS phase. The influence of raising both PBS and PE–g–MA contents on the phase morphology, mechanical and thermal properties was investigated and discussed.

## 2. Materials and Methods

### 2.1. Materials

LDPE, Etilinas Petlin C150Y (density = 0.92 g/cm^3^, melt flow index = 5 g/10 min at 230 °C and 2.16 kg) and PBS, FZ91PM (density = 1.26 g/cm^3^, melt flow index = 5 g/10 min at 230 °C and 2.16 kg) were supplied by Titan Polyethylene (M) Sdn. Bhd. and PTT Public Limited, Thailand, respectively. Polyethylene–graft–maleic anhydride compatibilizer (viscosity = 500 cP at 140 °C, density = 0.9 g/cm^3^ at 25 °C) [41] was purchased through Sigma-Aldrich Co. Ltd., UK. In the following text, it is denoted as PE–g–MA.

### 2.2. Preparation and Processing

#### 2.2.1. Blend Preparation

The sample was prepared according to the described procedure by Ferri et al. [42]. First, all of the materials were dried for 24 h in an oven at 60 °C to avoid trapped bubbles before melt mixing. Then, LDPE, PBS and PE–g–MA were pre-mixed in a zipper bag to promote preliminary homogenization. The compositions of the blends with specific sample codes are represented in Table 1. The desired compositions were processed in a Brabender internal mixer, which operated at 120 °C under a rotational speed of 50 rpm for 15 min [43]. After blending, the samples were cooled at room temperature.

#### 2.2.2. Compression Moulding

LDPE/PBS blends with and without compatibilizer were moulded by compression utilizing an electrical-heated hydraulic press. The hot press was performed at 120 °C with preheating time of 6 min and then 4 min of pressing at the same temperature. All compression-moulded sheets (150 × 150 × 1 mm) were then cold pressed for 6 min.

### 2.3. Characterization

#### 2.3.1. Fourier-Transform Infrared Spectroscopy (FTIR)

The structural analysis of the produced blends was identified by recording infrared spectra using an FTIR PerkinElmer spectrometer in a 400–4000 cm^−1^ wavelength range at room temperature. Pieces of the samples were placed on the sample holder and the obtained data were interpreted to determine the functional groups in the sample.

#### 2.3.2. Mechanical Analysis

Tensile tests were carried out in compliance with the ASTM D638 standard, using a universal testing machine (Shimadzu) with a load cell of 20 kN and a crosshead speed of 50 mm/min [38]. The compression-moulded sheets were used to cut dumbbell specimens with dimensions of 115 mm length, 19 mm width and 1 mm height. Five replicates of each blend composition were evaluated and the measurements of tensile strength (TS), elongation at break (EB) and Young’s modulus were recorded.

#### 2.3.3. Scanning Electron Microscopy (SEM)

The evaluation of blend morphology and interfaces using the ZEISS scanning electron microscopy with energy-dispersive X-ray (SEM-EDX) SUPRA 55VP model was carried out on the fracture surfaces of the samples (after mechanical analysis). The samples were gold-sputtered before observation to prevent electrical discharge. The accelerating voltage was kept at 10 kV.

#### 2.3.4. Thermogravimetry (TGA)

To determine the initial and maximum decomposition temperature of the produced blends, thermogravimetric analysis was characterized using a Mettler Toledo TGA/SDTA 851. The research was conducted at a 20 °C/min heating rate, with temperatures ranging from 25 to 600 °C. An atmosphere of nitrogen gas with a flow rate of 50 mL/min was used to heat a sample of 6 mg of the produced blends in the sample pan.

#### 2.3.5. Differential Scanning Calorimetry (DSC)

Differential scanning calorimetry analysis was analysed with a Mettler Toledo DSC under 50 mL/min of nitrogen flow. The sample weights were 8–10 mg. Two cycles of heating that covered intervals from −60 °C to 350 °C, with a heating/cooling rate of 10 °C/min, were performed. The heating and cooling regime comprised:

- First heating cycle from −60 °C to 350 °C; stabilization at 350 °C for 3 min;
- Cooling from 350 °C to −60 °C; stabilization at 350 °C for 3 min;
- Second heating cycle from −60 °C to 350 °C; stabilization at 350 °C for 3 min.

The melting temperature (T_m_) and crystallization temperature (T_c_) were evaluated after the melting and crystallization processes. The peak of the endothermic curve, T_m_ and the exothermic curve, T_c_ were used as reference points.

## 3. Results and Discussion

### 3.1. Fourier-Transform Infrared Spectroscopy (FTIR)

**Figure 1:**
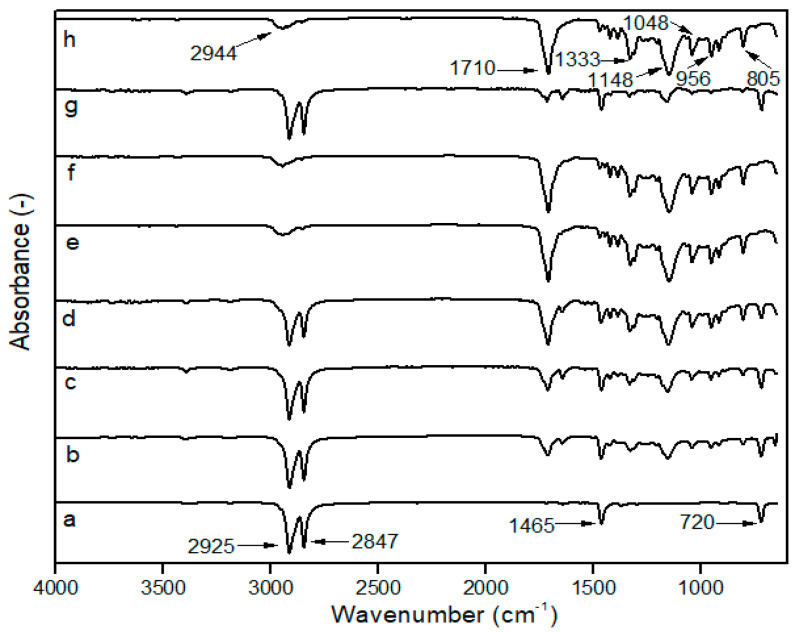
FTIR spectra of (**a**) neat LDPE; (**b**) 90LDPE/10PBS; (**c**) 80LDPE/20PBS; (**d**) 70LDPE/30PBS; (**e**) 60LDPE/40PBS; (**f**) 50LDPE/50PBS; (**g**) 90LDPE/10PBS/7PE–g–MA; (**h**) neat PBS.

The impact of mixing towards the interaction and functional groups was identified using FTIR. There will be no discernible differences in the FTIR spectra if two polymers formed completely immiscible blends with regard to the incorporation of each component. Conversely, when two polymers are miscible, a chemical interaction occurs between the chains of two different polymers, changing the blend’s IR spectra [16]. As a result, FT-IR spectroscopy can provide information about the potential interaction of LDPE and PBS with or without PE–g–MA, as shown in Figure 1.

The pure LDPE film displays two significant peaks of symmetric and asymmetric -CH_2_ at 2847 and 2925 cm^−1^, respectively. The peak at 1465 cm^−1^ was attributed to the bending vibrations of -CH_2_, whereas the peak at 720 cm^−1^ was related to the rocking vibrations of -CH_2_ [44,45]. PBS showed typical functional characteristics of the functional group at 2944 and 1333 cm^−1^, associated with symmetric and asymmetric deformational vibrations from -CH_2_ groups in the PBS main chains. The stretching vibration of the ester carbonyl (C=O) group was at 1710 cm^−1^. A strong -C-O-C stretching peak was seen at 1148 cm^−1^, corresponding to an ester linkage and the peak at 1044 cm^−1^ represents the -O-C-C-stretching vibration. Other significant peaks obtained are 956 and 805 cm^−1^, which were ascribed to C−OH bending in the carboxylic acid group [46,47]. These bands served as PBS indicators and the intensity increased with higher PBS content. The PE–g–MA spectrum exhibits characteristic peaks through -CH stretching vibration peaks of polyethylene at 2914 and 2847 cm^−1^, and C=O peak of maleic anhydride groups by 1705 and 718 cm^−1^ [48]. These confirmed that PBS and PE–g–MA were effectively incorporated into the LDPE matrix.

Moreover, the characteristic bands of the LDPE and PBS were visible in the spectra of LDPE/PBS blends. The polymer ratios influenced the intensity of absorption peaks. The intensity peaks (C=O, -C-O-C, -O-C-C- and C-OH) of PBS increased with higher PBS contents; meanwhile, the peak intensity of the CH_2_ group for LDPE reduced as PBS contents increased. As reported by Bumbudsanpharoke et al. [15], the intensity of PBS bands decreased with reduced PBS ratios after mixing with LLDPE. The blending of LPDE and PBS with the incorporation of PE–g–MA showed no formation of new peaks. No chemical reaction occurred between LDPE and PBS due to the absence of any new peak. The results were consistent with the HDPE/PBS/HDPE–g–MA blends according to El-Rafey et al. [29].

### 3.2. Mechanical Analysis

**Figure 2:**
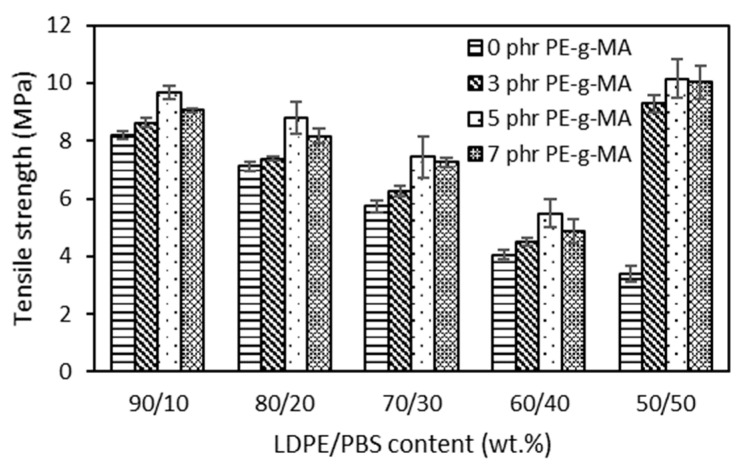
The effect of PBS content on tensile strength of uncompatibilized and compatibilized LDPE/PBS blends.

The effects of the different contents of PBS in the LDPE matrix are presented. Figure 2 shows that LDPE exhibited a typical ductile behaviour with high elongation at break (EB) of 447.21%, tensile strength (TS) of 12.42 MPa and a low elastic modulus of 245 MPa. On the other hand, PBS is a material that tends to be stiff and relatively brittle, characterized by a TS of 6.41 MPa, elastic modulus of 1109 MPa and a low EB with a value of 1.54%. After the physical blending of LDPE with PBS, the TS of the blends dropped monotonically as the PBS content became higher. These outcomes were expected and can be explained by the fact that the obtained blends led to non-homogenous structures and decreased polymer adhesion, which resulted in void spaces that decreased network strength [49]. The same result (6.42 MPa) was observed in the case of LDPE/PLA (80/20) compared to pure LDPE with 13.3 MPa [50] and LLDPE/PHB (80/20), which exhibited a low value (10.0 MPa) as compared to the LLDPE reference (16.6 MPa) [16] due to the immiscibility of the blends and poor interfacial adhesion between two polymers. Therefore, the decrease in tensile properties seems to reflect the low degree of miscibility and minimal interfacial contact among the two polymeric components of these blends, as discovered in morphological characterization.

The compatibilized LDPE/PBS blends presented higher TS than that of uncompatibilized LDPE/PBS blends. The LDPE/PBS/PE–g–MA blend showed higher TS at 5 phr of PE–g–MA. This might be attributed to strong interfacial adhesion as well as improved dispersion of PBS particles with the incorporation of PE–g–MA, resulting in better stress transfer among the matrix and the dispersed phase of the studied system [38]. The same effect was observed for HDPE/PBS blends with the incorporation of PE–g–MA [29]. They further explained that the increased TS was related to the physical interaction between MA groups on PE–g–MA and with hydroxyl and carboxyl groups of PBS and that physical entanglement exists among the PE chains in the PE–g–MA and LDPE matrix chains. The results showed that the TS of LDPE/PBS blends were improved with the increment of PE–g–MA up to 5 phr; however, it was reduced by incorporating beyond 5 phr of PE–g–MA in the blends. This indicated that 5 phr of the compatibilizer was sufficient to blend the PE–g–MA with polar polymer, PBS in the binary blends, as that content ultimately improved the structure.

Moreover, the addition of 7 phr of the PE–g–MA causes gradual structure weakening in the blends with the formation of agglomeration that promotes lower interfacial adhesion between the phases and subsequently decreases the TS [51]. The introduction of PE–g–MA of 3 phr did not strongly affect the TS of 60–90 wt.% LDPE blends. Most likely, the presence of 3 phr did not prove adequate to significantly affect the strength of the LDPE–PBS interfaces. The findings were similar to those obtained by Quitadamo et al. [52] in the case of TS for HDPE/PLA/wood flour composites when a compatibilizer was incorporated. Surprisingly, the TS for the compatibilized 50LDPE/50PBS blend gave higher TS and was almost similar to the TS obtained for neat LDPE, corresponding with a co-continuous structure and more homogenous structure due to the formation of partial miscibility blends. It should be noted that the enhancement in mechanical properties of the blends not only depends on the interface area improvement but is also correlated to other aspects, including the size and homogeneity of the dispersed phase and component intrinsic properties [44].

**Figure 3:**
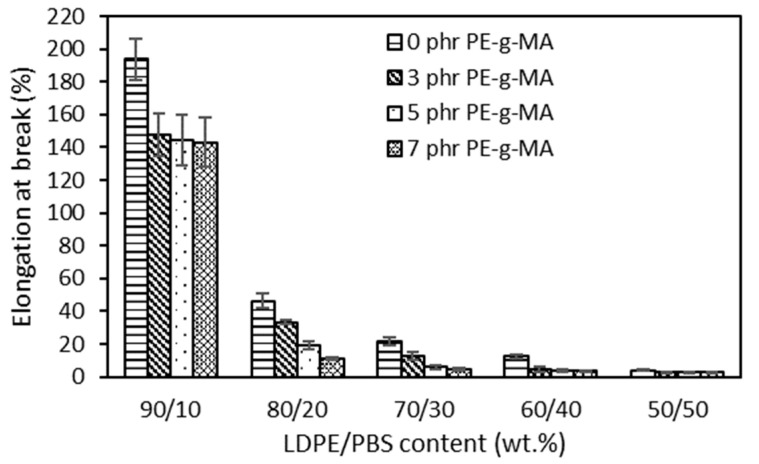
The effect of PBS content on elongation at break of uncompatibilized and compatibilized LDPE/PBS blends.

Figure 3 presents the elongation at break of both uncompatibilized and compatibilized LDPE/PBS blends. As the content of PBS gradually increased, EB decreased continuously due to the stiffness of PBS, which prevents the LDPE polymeric chains from moving freely and causes weak interfacial adhesion, voids and immiscibility between the phases [14]. The low EB value obtained for the blend reinforced with 50 wt.% PBS shows a brittle character compared to the neat LDPE. This can be explained by the rigid character of PBS. For the PP/polybutylene terephthalate (PBT) blend (70/30), about 10.0% EB of the blend was recorded as compared to 400% of pure PP [53]. The same results were observed in the majority of blends, such as LLDPE/PBS [15], LDPE/PLA [54] and PP/LDPE/PLA blends [55]. A ductile–brittle transition of the blends’ behaviour was used to explain this significant reduction. It was explained that brittle material, such as PBS, fractures without significant deformation when subjected to stress, whereas the capacity of a solid material to deform under stress is known as ductility, such as LDPE (Figure S1 and Table S1) [56]. At similar PBS content, the EB of compatibilized blends was reduced as compared to the uncompatibilized blends. This might clarify that the existence of PE–g–MA improved adhesion at the interface between LDPE and PBS. The reduction in EB, as shown by all blends, indicates a good degree of interfacial compatibility among polyolefin–polyester. In evaluating the impact of the PE–g–MA compatibilizer on LDPE/corn stalk (CS) composites, Ismail et al. [57] revealed that at similar CS content, the compatibilized composites had a lower EB in contrast to the uncompatibilized.

**Figure 4:**
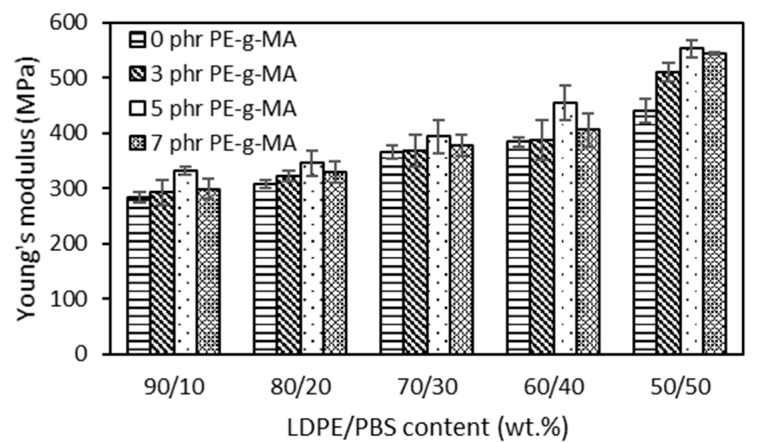
The effect of PBS content on the Young’s modulus of uncompatibilized and compatibilized LDPE/PBS blends.

The outcomes portrayed in Figure 4 showed that the Young’s modulus of both uncompatibilized and compatibilized blends became higher as the PBS content increased. PBS recorded higher modulus as compared to LDPE, so the addition of PBS to LDPE yielded blends with a higher modulus. These results were expected and can be explained by the obtained blends being more brittle due to the combined rigid character of PBS. Babaniyi et al. [58] demonstrated that the modulus of the used polyethylene (UPE)/polyhydroxybutyrate (PHB) was higher in line with PHB content, which was caused by the rigidity of PHB. The same result (2040 MPa) was observed in the case of HDPE/PLA (70/30) compared to the pure HDPE (890 MPa) [59] and LDPE/PLA blend (70/30), which presented a doubled value (420 MPa) with respect to the pure LDPE (200 MPa) [50]. The results also showed an increased modulus for the LDPE/PBS blend with PE–g–MA, which corresponds to an increased stiffness with respect to the uncompatibilized blends. PE–g–MA was added to highlight issues related to dispersion, other than improving the modulus of blends by enhancing adhesion across the interface [57]. Czarnecka-Komorowska et al. [41], who examined the effect of PE–g–MA, reported that incorporating PE–g–MA into the polyethylene/polyamide blends improved partial miscibility and compatibility between components, thus, enhancing the modulus of the blends. The addition of PE–g–MA in the blends was optimum at 5 phr. Beyond 5 phr, the modulus dropped due to the bad interfacial adhesion between the phases as a consequence of agglomeration on the excessive use of higher compatibilizer content. The same phenomenon was reported by Chow et al. [60]. The findings of this research also corroborated those of Djellali et al. [61] for LDPE and PLA blends, in which an increase in PLA content in the blends increased the TS and modulus but reduced the EB.

### 3.3. Scanning Electron Microscopy (SEM)

**Figure 5:**
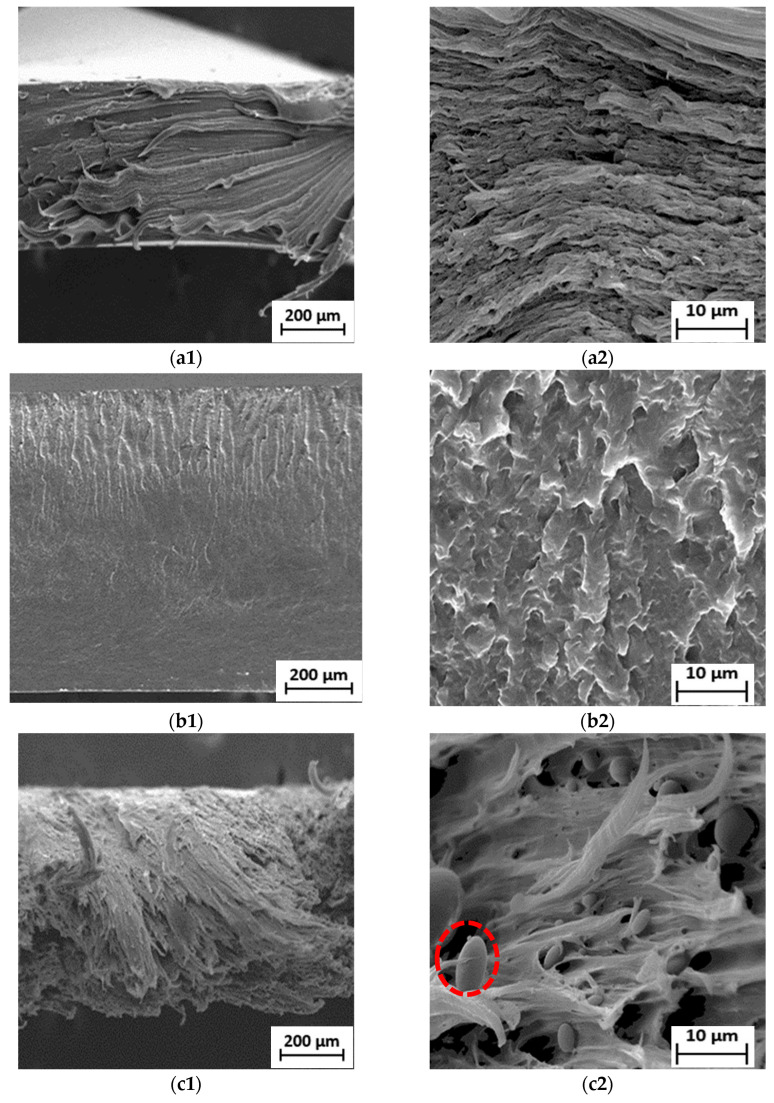

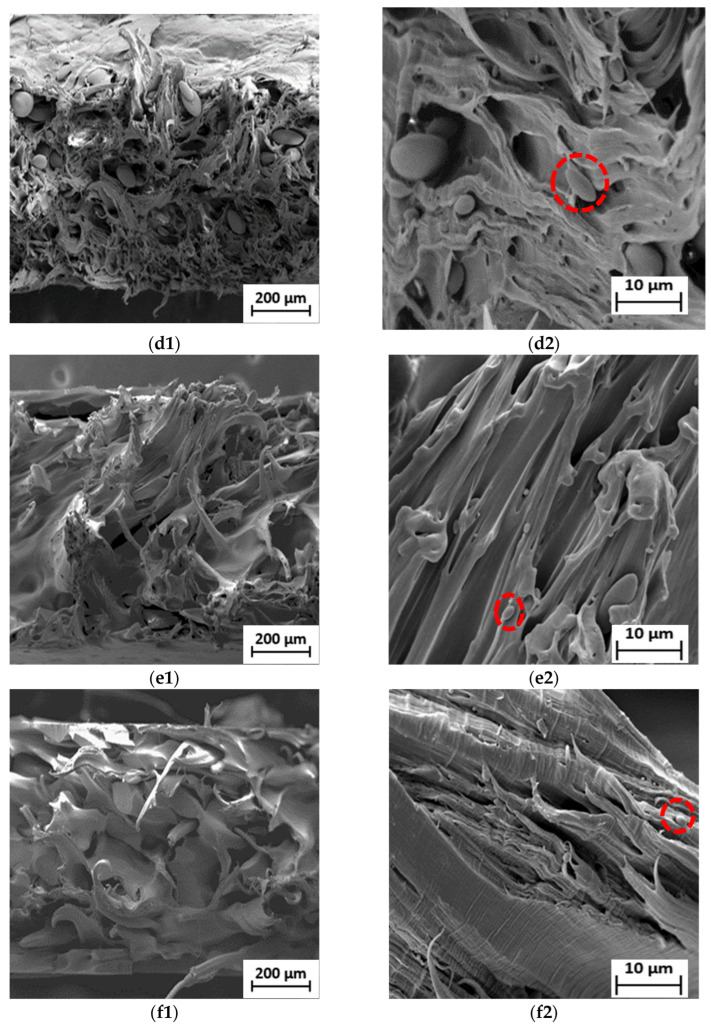

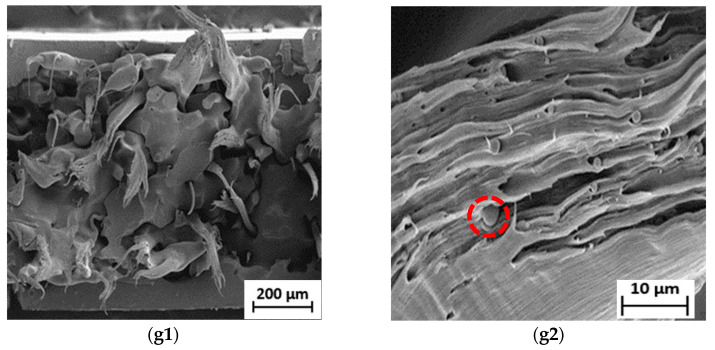
SEM micrographs of fractured surface of (**a1**) neat LDPE at 50× and (**a2**) neat LDPE at 1000×; (**b1**) neat PBS at 50× and (**b2**) neat PBS at 1000×; (**c1**) 90LDPE/10PBS at 50× and (**c2**) 90LDPE/10PBS at 1000×; (**d1**) 80LDPE/20PBS at 50× and (**d2**) 80LDPE/20PBS at 1000×; (**e1**) 70LDPE/30PBS at 50× and (**e2**) 70LDPE/30PBS at 1000×; (**f1**) 60LDPE/40PBS at 50× and (**f2**) 60LDPE/40PBS at 1000×; (**g1**) 50LDPE/50PBS at 50× and (**g2**) 50LDPE/50PBS blends at 1000× magnification.

SEM analysis is essential to determine the blends’ morphological features. Heterogeneous structures were predicted from the binary blends, in which the particles of PBS dispersed in the LDPE matrix. These particles’ size provides information about the interactions between the blend components, in which bigger particles indicate poor interactions and smaller particles show better interactions [54]. Figure 5 shows images obtained from the sample surface after mechanical analysis. The neat LDPE shown in Figure 5a exhibited the characteristics of a ductile material, such as plastically deformed behaviour and the presence of numerous tear lines. When a polymer begins to deform plastically in response to applied stress, crazing or shear yielding will occur. In contrast, the neat PBS depicts brittle material as having a relatively smooth fragmented surface of crazing with minimal plastic deformation, as illustrated in Figure 5b. The same behaviour was seen by Vrsaljko et al. [62] for the blend of PLA and LDPE.

It is commonly known that the weight ratio of the blending components primarily determines which of the two components created the matrix phase and the dispersed phase. In this case, the matrix phase represents the polymer with the largest proportion in the blend (i.e., LDPE) and the dispersed phase was found as spherical domains (red circles), ranging from 3 to 10 µm, corresponding to PBS. Figure 5c–g show the voids formed during fracture, which means that the weakly bound PBS dispersed phase was pulled out from the polyethylene matrix, promoting low TS properties. This finding shows that there is no particular interaction between PBS and LDPE phases. The fracture that occurred at the particle–matrix interface of the phases can be related to the weak interfacial adhesion between two components. The results obtained by Milovanovic and collaborators [63] confirm the blend’s immiscibility, which agrees with the observation in this work. The lack of miscibility between these polymers also caused the uneven dispersion of PBS in the LDPE matrix and the non-homogenous fractured surface. This is consistent with the fact that LDPE is composed of a hydrophobic ethylene chain (non-polar polymer), while PBS has oxygen atoms with functional groups of carbonyl and ester (polar polymer), indicating its immiscible characteristics [15]. Bhasney et al. [64] found that many voids were noticed because of poor adhesion between PLA and LLDPE. This morphology showed highly immiscible blends, owing to the high polarity differences between those of the two polymers.

The 90LDPE/10PBS blend shows higher plasticity deformation with pulled-out fibrils compared to the other blends. This proved that the addition of 10 wt.% PBS into the LDPE matrix did not significantly change the morphology of LDPE and resulted in the highest flexibility properties. The long fibrils were evidence of the highest EB of the blend. It was clarified that the ductile-type failure is defined by the formation of fibrils, and the length of the fibrils reveals how much the material deformed during failure. In contrast, the creation of voids indicates the poor interaction between LDPE and PBS phases [65]. The observations were in accordance with the literature published by Panrong et al. [66], who found that the LLDPE–bioplastic blend created cavities and fibrous-like morphology as a result of incompatible network. Due to the higher PBS content in the LDPE/PBS blends, lower interaction between LDPE and PBS could be seen. Increasing the PBS content caused a rough fractured surface with large voids and cavities as a result of their incompatibility. This result agrees with Aishah et al. [67], who investigated the recycled HDPE/PLA blend and found that the fracture of the blend formed a rough surface due to completely incompatible recycled HDPE with PLA, subsequently reducing the TS of the binary system. Blends containing 40 and 50 wt.% of PBS portrayed shorter fibrils in contrast to the neat and other blends. The failure mode changed from ductile to brittle as PBS content increased, which might be explained by the observation.

Surprisingly, the LDPE/PBS blends (60/40 and 50/50) exhibited a co-continuous structural morphology as PBS content increased. The domains of those blends are less noticeable, owing to the improved interfacial adhesion to the matrix phase of LDPE. Compared to the other blends, a homogenous fractured surface was discovered, demonstrating the coexistence of the two polymer phases as a co-continuous morphological structure [68]. The outcomes showed better molecular adhesion, which implied that the partial miscibility blends were achieved with the incorporation of 40 and 50 wt.% PBS. However, voids were still visible in these fractured blend surfaces. Recent research also demonstrated that a co-continuous structure was feasible to achieve. The structure of LDPE/TPS blends changed from a dispersed to a co-continuous structure at higher TPS (60 and 70 wt.%) concentrations as the blend’s viscosity ratio increased [69]. The current findings were also in agreement with Lu et al. [70], where the 50HDPE/50PLA blend formed co-continuous phase morphology in contrast to the other blends (80/20 and 60/40). Meanwhile, the morphological evaluation of the current research proved the immiscibility of LDPE/PBS blends at 10–30 wt.% PBS. The incorporation of 10 wt.% PBS showed a significant dispersion phase in the matrix phase as compared to the neat LDPE, while the addition of up to 50 wt.% PBS formed a co-continuous morphology, which represents the partially miscible blend. Higher PBS ratio resulted in a reduction in PBS particle size. As shown in Figure S2, the presence of oxygen (5.6%) corresponded to PBS, while a higher percentage of carbon (94.4%) corresponded to the LDPE matrix phase, which was similar to the outcome obtained for the blends of HDPE and PLA [14]. Direct mixing of LDPE/PBS blends without the addition of compatibilizers led to incompatible blends; subsequently, inferior mechanical properties were obtained.

**Figure 6:**
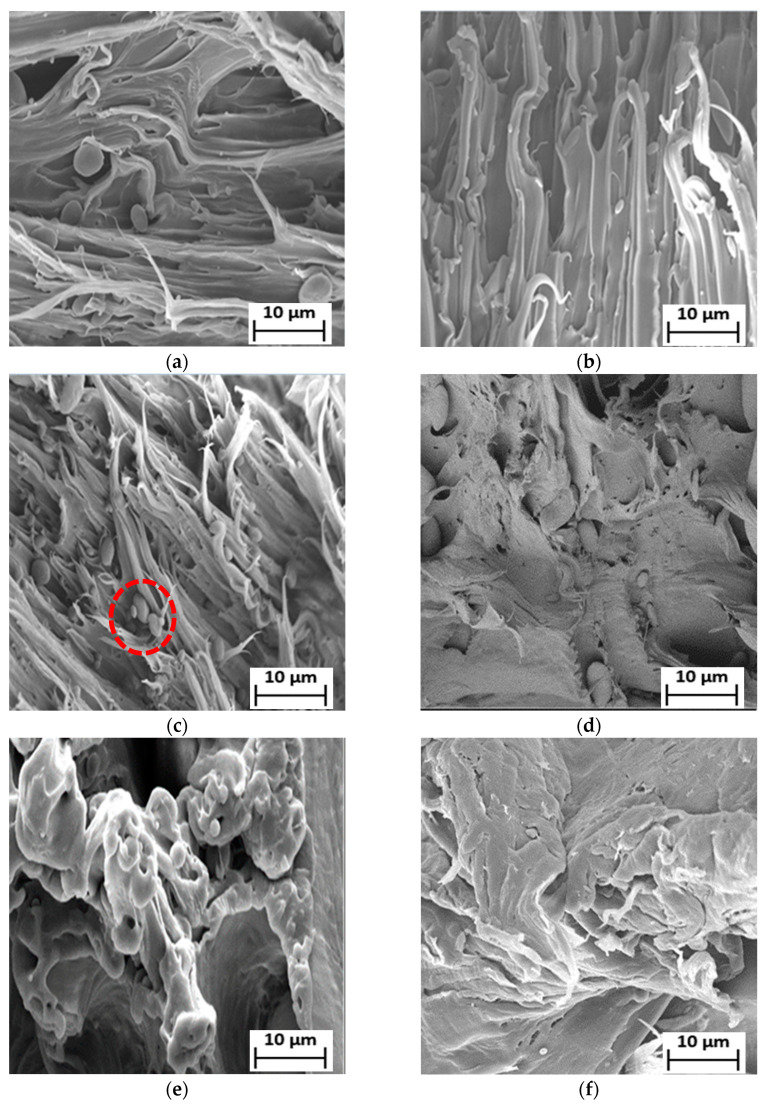

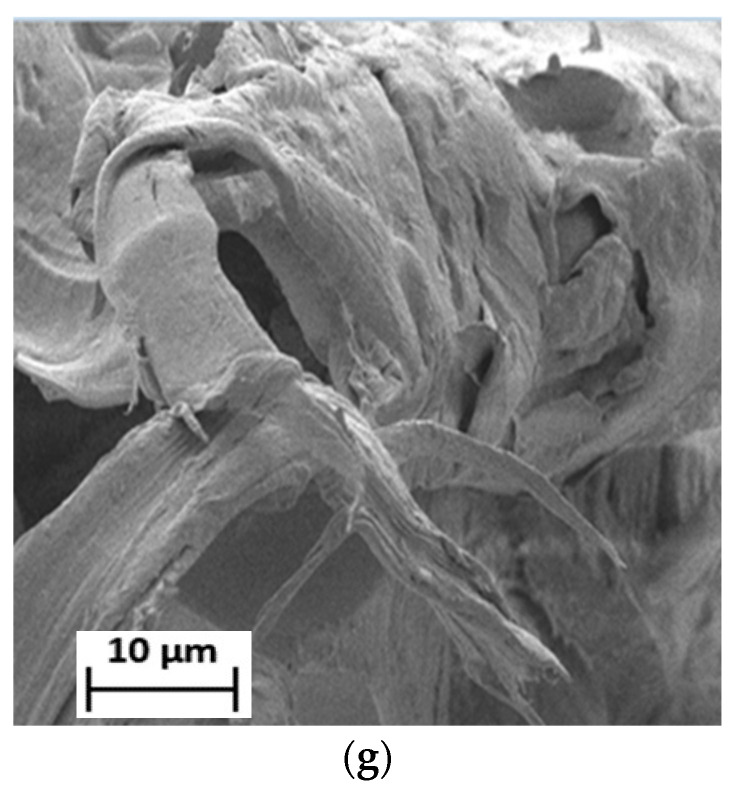
SEM micrographs of the fractured surface of (**a**) 90LDPE/10PBS/3PE–g–MA (**b**) 90LDPE/10PBS/5PE–g–MA (**c**) 90LDPE/10PBS/7PE–g–MA (**d**) 80LDPE/20PBS/5PE–g–MA (**e**) 70LDPE/30PBS/5PE–g–MA (**f**) 60LDPE/40PBS/5PE–g–MA and (**g**) 50LDPE/50PBS/5PE–g–MA blends at 1000× magnification.

For compatibilized LDPE/PBS blends, the introduction of 3–5 phr PE–g–MA compatibilizer reduced the dispersed-phase particles, varying in size from 2 to 5 µm, as observed from Figure 6a–g. The smaller-sized PBS particles were uniformly distributed within the matrix. These findings demonstrated that the PE–g–MA might improve the interfacial adhesion among the LDPE phase and PBS phase, resulting in good compatibility between these two polymers and, thus, more uniform dispersion of PBS in the LDPE/PBS blends. This effect might be brought about by the interaction and tendency of the compatibilizer to remain at the interface, which would reduce the interfacial tension and avoid domain coalescence [71]. This would be the primary factor behind the improvement in TS, as previously demonstrated in mechanical analysis. According to Moreno and Saron [72], the particle size of polyamide 6 domains was reduced because of strong interfacial adhesion to the continuous phase of LDPE containing 2.5 and 5 wt.% of PE–g–MA in the blends.

Additionally, the size and formation of voids in the fractured surface of the blends were also reduced in PE–g–MA for all blends. The compatibilized blends of 50LDPE/50PBS and 60LDPE/40PBS formed a co-continuous surface, but the presence of voids revealed that these two polymers were partially miscible with one another in contrast to the other compatibilized blends. In addition, there was no appearance of PBS particles in the LDPE matrix. A similar finding was described by other researchers about the polyolefin–polyester blend with partial miscibility morphology when a compatibilizer was incorporated [40]. The incorporation of 7 phr PE–g–MA compatibilizers in the blend causes the formation of agglomerates (red circle) of the PBS particles (Figure 6c). The increment content of PE–g–MA led to agglomeration, which caused lowered interfacial adhesion among the phases [60]. Therefore, this outcome was consistent with the data obtained from mechanical strength, where the blends with the incorporation of 5 phr PE–g–MA demonstrated the optimal TS with respect to the 7 phr PE–g–MA. The reduced miscibility of the blend [73] resulted from the combination of LDPE with a greater M_w_, 126,000 g/mol [74] and PBS with a lower M_w_, 65,000 g/mol [75]. The aggregation of compatibilizer increased as the content of PE–g–MA exceeded 5 phr due to the higher M_w_ of PE–g–MA, 31 200–112,500 g/mol [76]. According to Gibson et al. [77], the polymer aggregation increases with M_w_ and the significant change to the morphology of the polymer blend is caused by M_w_. In conclusion, PE–g–MA mainly enhanced the adhesion between the phases and increased the homogeneity of the blends by stabilizing their morphology in contrast to the uncompatibilized blends. The inclusion of PE–g–MA improves the interfacial adhesion due to the interaction between the maleic anhydride (of PE–g–MA) and ester linkage (of PBS), as previously reported by Darshan et al. [78].

### 3.4. Thermogravimetry (TGA)

**Figure 7:**
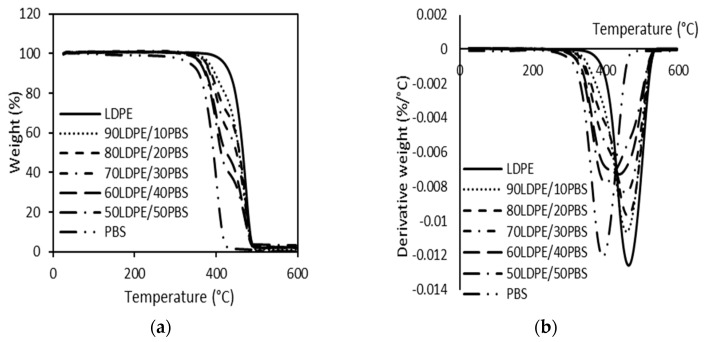
TGA (**a**) and DTG (**b**) curves of neat LDPE, neat PBS and relative LDPE/PBS blends.

The TGA and corresponding derivative TGA (DTG) curves of LDPE/PBS blends are presented in Figure 7a,b, respectively. Table 2 summarizes the results of the onset degradation temperature (T_onset_) and the maximum degradation temperature (T_max_). The thermal degradation of neat LDPE and PBS was different and there was only a single-step degradation process. It was shown that pure polymers demonstrated a single-step degradation, about 400–500 °C for LDPE [79,80] and 300–400 °C for PBS [81]. It is noted that the degradation of polyethylene is related to the C-C backbone scission reactions caused by the oxygen with the formation of radicals that can decompose easily, generating oxidized species [45]. According to Chrissafis et al. [82], the major degradation of PBS can be attributed to a random cleavage of the ester bonds caused by the ß-CH hydrogen transfer, leading to the creation of carboxylic end groups and vinyl groups.

For uncompatibilized LDPE/PBS blends, the T_onset_ and T_max_ were shifted to lower temperatures as the content of PBS became higher. The T_onset_ of PBS was about 331.4 °C, while that of LDPE was 405.4 °C. The LDPE/PBS blends displayed degradation curves between those of LDPE and PBS. The present finding seems to be consistent with the outcomes obtained by Thongsong et al. [83]. They found that both the T_onset_ and degradation temperature (T_d_) of PET/PBAT blends were continuously reduced. They ranged between the neat PET and neat PBAT as PBAT content increased due to the fact that PBAT is less thermally stable than PET. Meanwhile, the T_max_ of PBS was 392.6 °C and for LDPE, it was 463.6 °C, as presented in Figure 7. This can be justified by the fact that LDPE has more heat stability than PBS, which was lowered. The thermal stability and degradation of LDPE were superior to PBS, which could be due to the presence of ester bonds in the PBS structure, which was more easily decomposed at a lower temperature as compared to the C-C bonds in the backbone of PE. This result is in agreement with Rodriguez et al. [17], who studied HDPE/PLA blends and found that the decomposition temperature of PLA was lower than that of HDPE. The same phenomenon was observed with the HDPE/PBS blends [78]. These results indicated that the thermal properties of biodegradable polymers (PBS and PLA) are less stable than the non-biodegradable polymer, such as PE.

**Figure 8:**
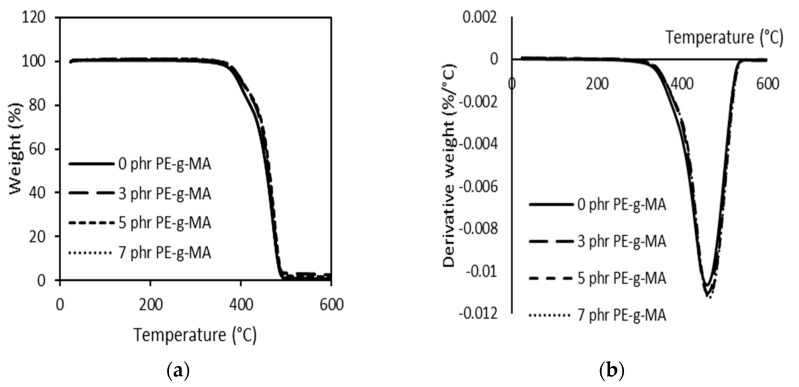
TGA (**a**) and DTG (**b**) curves of 90LDPE/10PBS blends with varying PE–g–MA content.

As seen in Figure 8a,b, the compatibilized blends presented similar behaviour to the LDPE/PBS blend. When 3–7 phr PE–g–MA was added, the T_onset_ and T_max_ increased from 388 to 399 °C and 459 to 463 °C, respectively. This means that the LDPE/PBS/PE–g–MA blends have higher thermal stability than LDPE and PBS. The significant increase in thermal stability was presented by 5 phr PE–g–MA. This might be the better dispersion of PBS in the LDPE matrix with the participation of the compatibilizer, thus, enhancing the interfacial adhesion between the dispersed phase and matrix phase. Darshan et al. [78] also reported the same effect for blends consisting of HDPE, PBS and the compatibilizer. Additionally, there was physical interaction involved among the blend components with the compatibilizer, and these required more energy and higher temperature to induce random chain scission through thermal degradation [84]. The result is consistent with what was found by Czarnecka-Komorowska et al. [41] with regard to PE–g–MA-incorporated LDPE/PBS blends. The authors noted that the incorporation of compatibilizer induced a significant decrease in the size of the polyamide (PA) domains, associated with the higher thermal stability of the PE/PA blends. Thus, the findings obtained in this study were in line with the results from mechanical analysis, in which the participation of PE–g–MA portrayed an excellent mechanical strength as compared to the uncompatibilized LDPE/PBS blends.

### 3.5. Differential Scanning Calorimetry (DSC)

**Figure 9:**
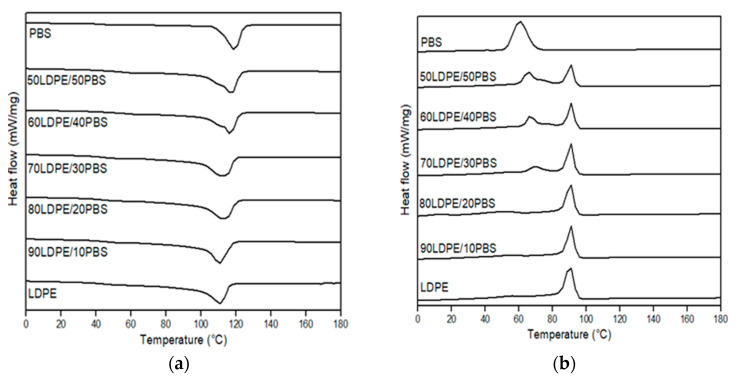
DSC thermograms of neat LDPE, neat PBS and relative LDPE/PBS blends. (**a**) First heating and (**b**) cooling scan.

**Figure 10:**
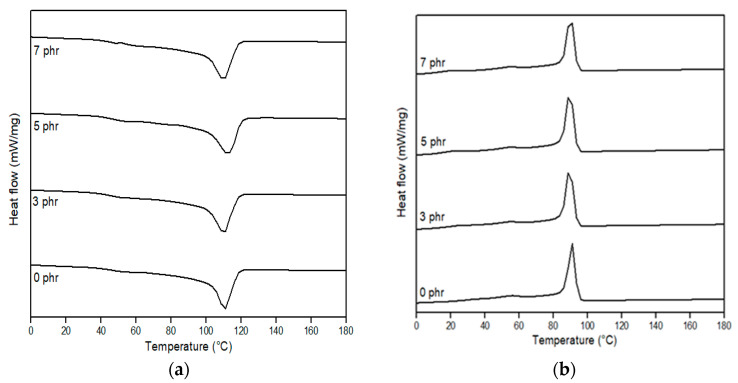
DSC thermograms of 90LDPE/10PBS blends with varying PE–g–MA content: (**a**) first heating and (**b**) cooling scan.

DSC analysis is an interesting way to investigate the miscibility of two polymers in terms of melting behaviour. In Figure 9 (neat polymer and uncompatibilized blends) and Figure 10 (compatibilized blends), illustrative DSC thermograms for the first heating and the cooling cycle are demonstrated, while the main thermal parameters in relation to melting temperature (T_m_) and crystallization temperature (T_c_) are summarized in Table 3. These findings showed that the glass transition temperature (T_g_) cannot be detected in all blends. The T_g_ of LDPE was not determined because the temperature range analysed is outside of the measuring range of the device. The T_g_ of PBS could not be determined from the DSC curve. The literature value of LDPE and PBS T_g_ is −110 °C and −31 °C, respectively [63,85].

Compared to the T_m_ value for neat LDPE (111.8 °C) and neat PBS (117.6 °C), the T_m_ values of 50LDPE/50PBS (117.3 °C) blends showed curves in between those of the neat components. Similar behaviour was described by Rodriguez et al. [17] for HDPE/PLA blends. LDPE/PBS polymer blends in a weight ratio of 70–90% LDPE have a single T_m_ peak representing the T_m_ of the LDPE phase without the presence of the T_m_ peak for PBS. This might be due to the overlapped peak between LDPE and PBS because the T_m_ value was closer between them. The same effect was shown by Hemsri et al. [36], where a single T_m_ peak was seen for blends of LDPE and PBAT because the intensity of the T_m_ peak of PBAT was relatively low and relatively overlapped with the T_m_ peak of LDPE. Meanwhile, the second peak intensity was more noticeable at increased PBS weight ratios (40 and 50 wt.%) with the formation of a shoulder peak at low temperature. The incorporation of PBS into the LDPE matrix did not show any changes in melting peak positions relative to the neat polymers, which was clear evidence of the weak interactions or lack of miscibility between these two polymers and in agreement with the SEM studies. The low miscibility between the dispersed phase and matrix phase was also observed through DSC characterization by Ferri et al. [42], Quiles-Carrillo et al. [86] and Quitadamo et al. [87], which presented the same behaviour as the results of this research. The appearance of two melting peaks of the produced blends and the dissimilarity in the T_m_ of the polymers before and after the blending confirmed the absence of any chemical interaction between components, as supported by the FTIR results [29]. The presence of two separate T_m_ peaks demonstrated the physical interaction between the components, even though co-continuous-phase morphologies were formed for 50/50 and 60/40 wt.% LDPE/PBS blends. Similar phenomena were also observed for T_c_ with two separated peaks. The T_c_ of LDPE was not affected by the presence of PBS, but the T_c_ of PBS for the blends reduced as PBS increased. This indicated that the PBS behaves as an inhibitor in the recrystallization process at a higher PBS ratio. The present finding seems to agree with Du et al. [88]. They found a decrease in T_c_ of composites due to the presence of graphene oxide, which reduced the molecular chain mobility and limits its rearrangement, causing a delay in the crystallization process.

From the data, no changes were observed in T_m_ for the 90LDPE/10PBS blend at 3 phr PE–g–MA in contrast to the 90LDPE/10PBS blend without compatibilizer because the participation of compatibilizer was not sufficient enough to improve the interfacial adhesion between the dispersed phase and matrix phase. DSC data revealed that the T_m_ of PE–g–MA was 101.1 °C (Figures S3 and S4). The outcome was consistent with the previous literature [89]. A similar trend was presented by Czarnecka-Komorowska et al. [41], who discussed the impact of compatibilizer on the melting behaviour of the polyamide compounded with polyethylene, where the addition of 1 wt.% PE–g–MA did not show any changes in T_m_ as compared to the PE/PA blend without compatibilizer. They found that the incorporation of PE–g–MA to the PE/PA blend at 3 wt.% resulted in a slightly higher T_m_ owing to the interfacial interaction between the blend and compatibilizer. About 5 phr of a compatibilizer seems to enhance the thermal properties (thermal stability) and the affinity between LDPE and PBS. Additionally, the T_m_ value decreased as the compatibilizer content was raised to 7 phr. The size reduction and more uniform dispersion of the PBS domain in the LDPE matrix influenced the increase in interfacial interaction between components. This statement was supported by the SEM analysis for compatibilized LDPE/PBS blends. The excess compatibilizer dispersed in the binary blends has a tendency to agglomerate, thus, reducing its T_m_. This explanation was in line with the justification given in another publication [51]. The presence of a single peak for T_m_ and T_c_ for LDPE/PBS/PE–g–MA was discussed above. However, the T_c_ values obtained for the LDPE/PBS/PE–g–MA blends were identical to those of the LDPE/PBS blend, indicating that the PE–g–MA diminished the influence of PBS on the crystallization of LDPE. This complies with the findings reported by Oner et al. [37].

## 4. Conclusions

The present study describes the preparation of LDPE/PBS blends with polyethylene–graft–maleic anhydride (PE–g–MA) by melt compounding at different ratios of PBS (10, 20, 30, 40 and 50 wt.%) and PE–g–MA (3, 5 and 7 phr), to develop a partially biodegradable and economically viable blend with homogenous structural, superior mechanical and thermal properties. The FTIR spectra confirmed that the LDPE, PBS and PE–g–MA were present separately in all of the produced blends. The incorporation of PBS into the LDPE matrix resulted in the substantial decrement of mechanical properties in relation to the TS and EB. This reduction observed for these properties explained a poor stress transfer between the two polymer phases due to the lack of incompatibility. The dispersed PBS phase potentially served as a stress concentrator in the LDPE matrix, promoting the sample deformation. SEM indicated that the LDPE was incompatible with the incorporation of 10–30 wt.% PBS, but the blends are partially miscible at 40–50 wt.% PBS content. The TGA analysis revealed that the inclusion of the PBS decreased the thermal properties of the binary blends, whereas the T_m_ of the blends did not change in contrast to the pure polymer, as observed by DSC. The thermal studies on the blends further proved the poor compatibility or lack of miscibility between LDPE and PBS. As the 90LDPE/10PBS blend showed excellent mechanical properties, this blend was considered for further investigations by introducing a PE–g–MA compatibilizer.

In an effort to enhance the miscibility, the impact of PE–g–MA in terms of morphology, mechanical and thermal properties of LDPE/PBS blends was investigated. The morphological study showed that the incorporation of PE–g–MA greatly enhanced the structure of the LDPE/PBS/PE–g–MA blends as compared to the LDPE/PBS binary blends, resulting in a reduction in PBS domain size of the dispersed phases and better interfacial adhesion. Adding 5 phr PE–g–MA to the LDPE/PBS blends increased the homogeneity of the blends owing to the interaction between the maleic anhydride (of PE–g–MA) and ester linkage (of PBS). Regarding the thermal analysis, it was noted that the addition of PE–g–MA up to 5 phr increased the interactions among polymer phases, subsequently improving the thermal properties of the blends in contrast to the uncompatibilized. To sum up, the incorporation of the PE–g–MA compatibilizer demonstrated a desirable approach to improve the miscibility between polyolefins and polyesters, as it can potentially contribute to the development of environmentally friendly polymer technologies. The fabrication of a partially biodegradable blend is an alternative for commercialising eco-products to reduce the dependency on non-biodegradable polymers. It can then be considered as an excellent candidate for application in the packaging industry, such as food trays, lids and storage containers.

## Figures and Tables

**Table 1:** Sample codes and compositions of uncompatibilized and compatibilized low-density polyethylene/poly(butylene succinate) (LDPE/PBS) blends.

Sample Code	LDPE (wt.%)	PBS (wt.%)	PE–g–MA (phr)
LDPE	100	-	-
PBS	-	100	-
90LDPE/10PBS	90	10	0
90LDPE/10PBS/3PE–g–MA	90	10	3
90LDPE/10PBS/5PE–g–MA	90	10	5
90LDPE/10PBS/7PE–g–MA	90	10	7
80LDPE/20PBS	80	20	0
80LDPE/20PBS/3PE–g–MA	80	20	3
80LDPE/20PBS/5PE–g–MA	80	20	5
80LDPE/20PBS/7PE–g–MA	80	20	7
70LDPE/30PBS	70	30	0
70LDPE/30PBS/3PE–g–MA	70	30	3
70LDPE/30PBS/5PE–g–MA	70	30	5
70LDPE/30PBS/7PE–g–MA	70	30	7
60LDPE/40PBS	60	40	0
60LDPE/40PBS/3PE–g–MA	60	40	3
60LDPE/40PBS/5PE–g–MA	60	40	5
60LDPE/40PBS/7PE–g–MA	60	40	7
50LDPE/50PBS	50	50	0
50LDPE/50PBS/3PE–g–MA	50	50	3
50LDPE/50PBS/5PE–g–MA	50	50	5
50LDPE/50PBS/7PE–g–MA	50	50	7

PE–g–MA is the maleic anhydride grafted polyethylene, phr (per hundred resin) denotes the weight parts of the compatibilizers incorporated to one hundred weight parts of the base LDPE/PBS blend.

**Table 2:** Results of thermogravimetric analysis of neat LDPE, neat PBS and relative LDPE/PBS blends.

Sample	Onset Degradation Temperature (°C)	Maximum Degradation Temperature (°C)
LDPE	405.4	463.6
90LDPE/10PBS	388.6	459.3
80LDPE/20PBS	383.9	461.9
70LDPE/30PBS	334.9	455.5
60LDPE/40PBS	332.0	438.4
50LDPE/50PBS	332.3	400.4
PBS	331.0	392.6
90LDPE/10PBS/3PE–g–MA	394.6	460.0
90LDPE/10PBS/5PE–g–MA	399.0	463.4
90LDPE/10PBS/7PE–g–MA	398.7	463.6

**Table 3:** Results of DSC tests of neat LDPE, neat PBS, uncompatibilized and compatibilized LDPE/PBS blends.

Sample	T_m_ LDPE (°C)	T_m_ PBS (°C)	T_c_ LDPE (°C)	T_c_ PBS (°C)
LDPE	111.8	-	90.0	-
90LDPE/10PBS	109.6	-	90.2	-
80LDPE/20PBS	112.1	-	89.7	-
70LDPE/30PBS	110.6	-	89.7	69.3
60LDPE/40PBS	110.8	117.4	90.2	66.7
50LDPE/50PBS	110.8	117.3	90.0	65.6
PBS	-	117.6	-	60.1
90LDPE/10PBS/3PE–g–MA	109.8	-	89.2	-
90LDPE/10PBS/5PE–g–MA	112.1	-	89.2	-
90LDPE/10PBS/7PE–g–MA	109.6	-	89.6	-

T_m_, melting temperature (first heating scan); T_c_, crystallization temperature (cooling scan).

## Data Availability

The data presented in this study are available on request from the corresponding author.

## Supplementary Materials

The following supporting information can be downloaded at: https://www.mdpi.com/article/10.3390/polym15020261/s1, Figure S1: Behaviour of ductile and brittle material; Table S1: Material deformation on mechanical analysis; Figure S2: (a) Scanning electron microscopy with (b) energy-dispersive X-ray (SEM-EDX) analysis of 80LDPE/20PBS blend at 1000× magnification; Figure S3: DSC thermogram: First heating scan of PE–g–MA; Figure S4: DSC thermogram: Cooling scan of PE–g–MA.
