# Supplementary material for: Blending of Low-Density Polyethylene and Poly(Butylene Succinate) (LDPE/PBS) with Polyethylene–Graft–Maleic Anhydride (PE–g–MA) as a Compatibilizer on the Phase Morphology, Mechanical and Thermal Properties
Source: Polymers (Basel). 2023 Jan 4;15(2):261. doi: 10.3390/polym15020261 (PMC9860711; doi:10.3390/polym15020261)
Supplement: Supplementary file 1 [file polymers-15-00261-s001.zip › polymers-2072328-supplementary.pdf]

SUPPLEMENTARY MATERIAL

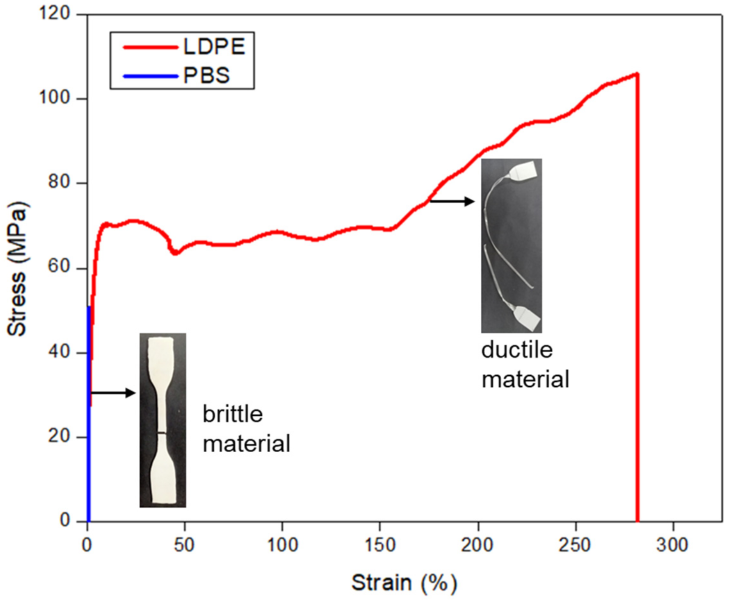

Figure S1. Behavior of ductile and brittle material

Table S1: Material deformation on mechanical analysis

Material	Before mechanical analysis	After mechanical analysis
LDPE	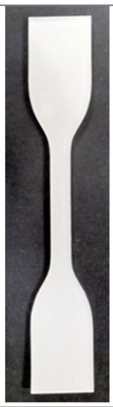	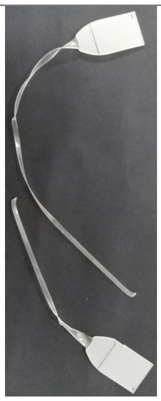
PBS	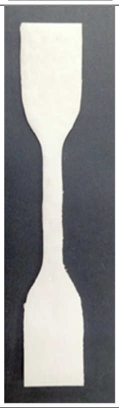	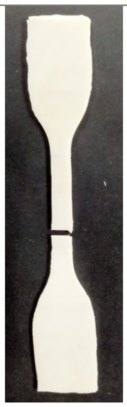

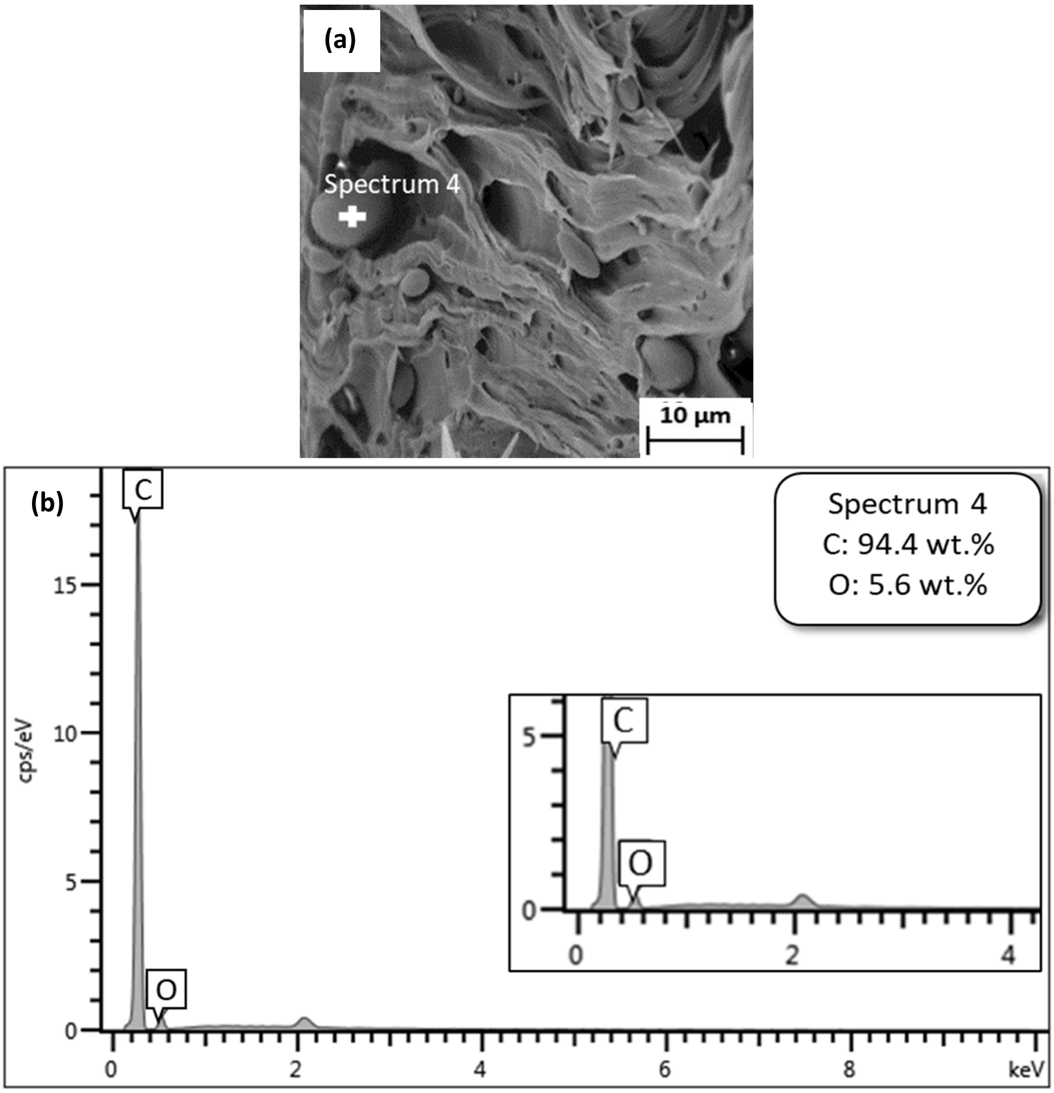

Figure S2: (a) Scanning electron microscopy with **(b)** energy-dispersive X-ray (SEM-EDX) analysis of 80LDPE/20PBS blend at 1000× magnification

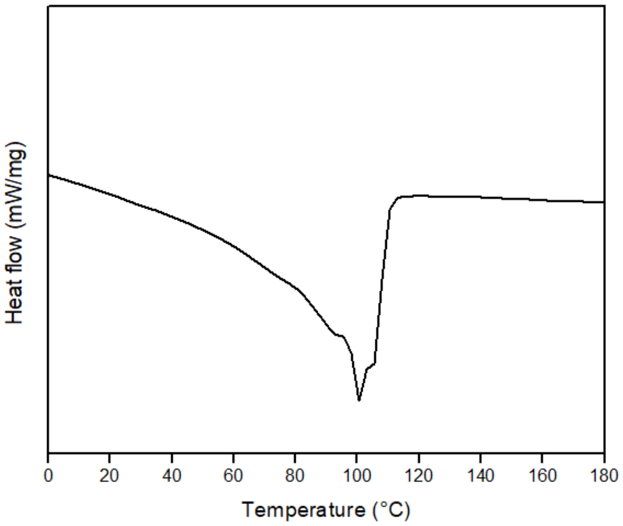

Figure S3: DSC thermogram: First heating scan of PE-g-MA

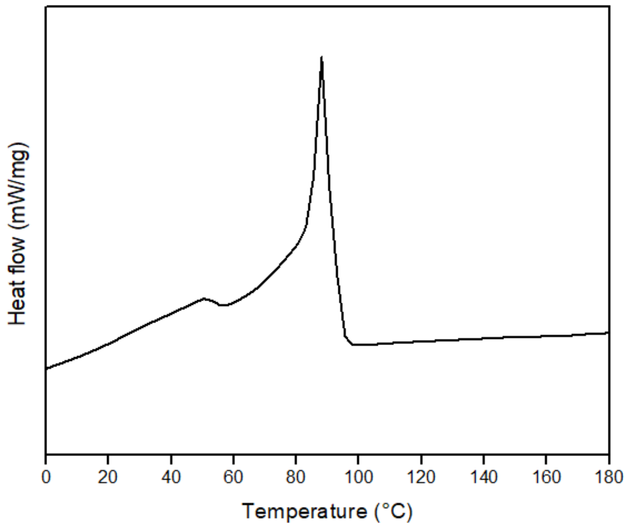

Figure S4: DSC thermogram: Cooling scan of PE-g-MA
